# Acid-sensing ion channel 3 decreases phosphorylation of extracellular signal-regulated kinases and induces synoviocyte cell death by increasing intracellular calcium

**DOI:** 10.1186/ar4577

**Published:** 2014-06-12

**Authors:** Weiyi Gong, Sandra J Kolker, Yuriy Usachev, Roxanne Y Walder, David L Boyle, Gary S Firestein, Kathleen A Sluka

**Affiliations:** 1 Department of Anesthesiology, Fujian Medical University Union Hospital, Fuzhou City, Fujian, China; 2 Department of Physical Therapy and Rehabilitation Science, Pain Research Program, University of Iowa Carver College of Medicine, 500 Newton Road, 1-248 Medical Education Building, Iowa City, IA 52242, USA; 3 Department of Pharmacology, University of Iowa Carver College of Medicine, Iowa City, IA 52242, USA; 4 Division of Rheumatology, Allergy and Immunology, University of California San Diego School of Medicine, La Jolla, CA, USA

## Abstract

**Introduction:**

Acid-sensing ion channel 3 (ASIC3) is expressed in synoviocytes, activated by decreases in pH, and reduces inflammation in animal models of inflammatory arthritis. The purpose of the current study was to characterize potential mechanisms underlying the control of inflammation by ASIC3 in fibroblast-like synoviocytes (FLS).

**Methods:**

Experiments were performed in cultured FLS from wild-type (WT) and *ASIC3-/-* mice, *ASIC1-/-* mice, and people with rheumatoid arthritis. We assessed the effects of acidic pH with and without interleukin-1β on FLS and the role of ASICs in modulating intracellular calcium [Ca^2+^]_i_, mitogen activated kinase (MAP kinase) expression, and cell death. [Ca^2+^]_i_ was assessed by fluorescent calcium imaging, MAP kinases were measured by Western Blots; ASIC, cytokine and protease mRNA expression were measured by quantitative PCR and cell death was measured with a LIVE/DEAD assay.

**Results:**

Acidic pH increased [Ca^2+^]_i_ and decreased p-ERK expression in WT FLS; these effects were significantly smaller in *ASIC3-/-* FLS and were prevented by blockade of [Ca^2+^]_i_. Blockade of protein phosphatase 2A (PP2A) prevented the pH-induced decreases in p-ERK. In WT FLS, IL-1β increases ASIC3 mRNA, and when combined with acidic pH enhances [Ca^2+^]_i_, p-ERK, IL-6 and metalloprotienase mRNA, and cell death. Inhibitors of [Ca^2+^]_i_ and ERK prevented cell death induced by pH 6.0 in combination with IL-1β in WT FLS.

**Conclusions:**

Decreased pH activates ASIC3 resulting in increased [Ca^2+^]_i_, and decreased p-ERK. Under inflammatory conditions, acidic pH results in enhanced [Ca^2+^]_i_ and phosphorylation of extracellular signal-regulated kinase that leads to cell death. Thus, activation of ASIC3 on FLS by acidic pH from an inflamed joint could limit synovial proliferation resulting in reduced accumulation of inflammatory mediators and subsequent joint damage.

## Introduction

Acid-sensing ion channels (ASICs) are the primary acid sensors in nociceptors, and substantial work shows that activation of acid sensing ion channels (ASIC1, ASIC3) contributes to the development of musculoskeletal pain [1-8]. However, we previously demonstrated localization of ASIC3 protein to Type B synoviocytes in mouse joint, and ASIC3 protein and mRNA in cultured fibroblast-like synoviocytes (FLS) [6,9]. Acidic pH in cultured FLS increases (Ca^2+^)_i_, and facilitates release of hyaluronic acid; these pH-dependent effects are reduced in FLS from *ASIC3-/-* mice [9].

Rheumatoid arthritis (RA) is a systemic inflammatory disease that particularly affects synovial joints. Acidic pH is associated with inflammation in rheumatoid joints where pH drops between pH 6.0 and 7.0 [10,11]. ASIC3 senses decreases in pH within the physiological range that would normally be found within an inflamed joint (pH 6.0 to 7.0) [5,12]. In RA, synoviocytes are key players in the production of inflammatory mediators and proteases that subsequently enhance the inflammatory process and joint damage [13-17]. Surprisingly, we found that *ASIC3-/-* mice have enhanced inflammation, despite reduced pain behaviors, in the collagen-induced arthritis model [1]. The enhanced inflammation is accompanied by increased joint destruction and inflammatory mediator production [1]. As inflammatory mediators and decreases in pH occur simultaneously in inflammatory arthritis, we further tested the effects of combining acidic pH with IL-1β - this combination results in cell death [1]. Thus, ASIC3 appears to play a protective role in joints. Although ASIC1 is expressed in FLS, the role of ASIC1 in FLS is unclear.

Increases in (Ca^2+^)_i_ have multiple effects on cells including modulation of intracellular messengers and promotion of cell death. The intracellular signaling molecules mitogen-activated protein kinases (MAPKs) in FLS are critical players in the inflammatory process in RA. MAPKs are activated by cytokines and Toll-like receptors in human FLS with a subsequent positive feedback loop that enhances expression of inflammatory cytokines [16-20]. For example, the MAPK c-Jun N-terminal kinase (JNK) increases MMP3 gene expression to increase cellular matrix degradation, which results in joint destruction [18,20-22]. *JNK2-/-* mice have modestly lower cartilage destruction, and inhibition with a non-specific JNK antagonist reduces expression and release of inflammatory cytokines [19,22]. MAPK activation, including extracellular signal-regulated kinase (ERK), JNK, and p38, can result in cell death in a variety of cell types including neurons, cancer, chondrocytes, and macrophages [23-26]. Interestingly, increased (Ca^2+^)_i_ enhances PP2A catalytic subunit expression which results in decreased ERK phosphorylation [27]. It is, therefore, possible that low pH activates ASIC3 to increase (Ca2+)_i_, which in turn reduces MAPK activation and promotes cell death. The purpose of the current study was to characterize potential mechanisms underlying the control of inflammation by ASIC3 in FLS, compared to wild-type (WT) and ASIC1 FLS. Specifically we tested if (1) ASIC1 and ASIC3 mediate acidic pH-induced increases in (Ca2+)_i_ in FLS; (2) acidic pH changes phosphorylation and expression of MAPK through ASIC1 and ASIC3; (3) effects of pH on (Ca2+)_i_ and MAPK are enhanced in the presence of the inflammatory cytokine IL-1β, and (4) increases in (Ca2+)_i_ drive the changes in MAPK activity and cell death.

## Methods

### Mice

C57Bl/6 J mice (WT), and congenic *ASIC3-/-* and AISC1-/- mice on a C57B1/6 J background were bred at the University of Iowa or the University of California San Diego Animal Care Facility. The *ASIC3-/-* and *ASIC1-/-* mice strains have been previously described and characterized in multiple studies [7,8,28,29]. Male mice, 9 to 10 weeks of age, were used in these studies. All experiments using animals were approved by Animal Care and Use Committee at the University of Iowa and the University of California San Diego, and conducted in accordance with National Institutes of Health (NIH) guidelines. Use of synoviocytes from human subjects was approved by the Institutional Review Board in the Human Subjects Office at the University of Iowa and in the Human Subjects Protection Program at the University of California San Diego.

### Fibroblast-like synoviocyte (FLS) isolation and culture

#### Preparation of FLS from mice

FLS were isolated from WT, *ASIC3-/-* and *ASIC1-/-* mice according to previously published methods [14,19]. Mice were euthanized with sodium pentobarbital (100 mg/kg, ip) and the limbs excised above the knee and elbow joints. After rinsing the excised limbs in ethanol, the knee, ankle and elbow joints were stripped of muscle and tendon. The remaining bone and tissue were finely minced and incubated in 0.5 mg/ml Type VIII collagenase (Sigma, St. Louis, MO, USA) in RPMI (Invitrogen, Carlsbad, CA, USA) at 37°C for 2 h. The tissue was pelleted at 1,200 rpm and the supernatant discarded. The pellet was rinsed twice in high-glucose DMEM (Gibco, Invitrogen, Carlsbad, CA, USA) supplemented with penicillin, streptomycin, 10% FBS, and 0.5% gentamycin (Cellgro, Manassas, VA, USA), resuspended, plated onto T75 culture flasks and incubated at 37°C with 5% CO_2_. FLS were grown until >80 to 90% confluent before passaging. FLS were at passage 3 to 4 for use in western blot, (Ca2+)_i_, mRNA expression studies, and Live/Dead assays.

#### Preparation of human synovial tissue and FLS

This study was approved by the Institutional Review Board of University of California, San Diego School of Medicine and informed consent was obtained from all participants. Synovial tissue was obtained from patients with RA at the time of total joint replacement or synovectomy as previously described. The diagnosis of RA conformed to American College of Rheumatology 1987 revised criteria. The samples were processed for cell culture. The synovium was minced and incubated with 1 mg/ml collagenase type VIII (Sigma) in serum-free RPMI 1640 (Gibco BRL, Grand Island, NY, USA) for 1 h at 37°C, filtered, extensively washed, and cultured in DMEM (Gibco BRL) supplemented with 10% FBS (Gemini Bio Products, Calabasas, CA, USA), penicillin, streptomycin, gentamicin, and glutamine, in a humidified 5% CO_2_ atmosphere. Cells were allowed to adhere overnight, non-adherent cells were removed, and adherent FLS were split at 1:3 when 70 to 80% confluent. FLS were used from passage 3 through 9 during which time they are a homogeneous population of cells (<1% CD11b-positive, <1% phagocytic, and <1% FcγRII- and FcγRIII-receptor-positive). FLS were cultured and used at 80% confluence. Cells were synchronized in 0.1% FBS for 24 h before the addition of the appropriate stimulus.

### Calcium imaging

External physiological pH solutions used for Ca^2+^ imaging contained 120 mM NaCl, 5 mM KCl,1 mM MgCl_2_, 2 mM CaCl_2_, 10 mM HEPES, and 10 mM MES; the pH of each solution was adjusted with tetramethylammonium hydroxide, and osmolarity was adjusted with tetramethylammonium chloride [30]. Cells were plated at 30,000 cells/dish in poly-L-lysine (Sigma) coated 35-mm glass-bottom petri dishes (MatTek Corp., Ashland, MA, USA), and grown for 24 h in 10% FBS, antibiotic-supplemented DMEM and subsequently serum-starved (0.1% FBS, antibiotic-supplemented DMEM) 24 h prior to testing.

After rinsing with pH 7.4 external buffer, cells were loaded with the Ca^2+^-sensitive fluorescent indicator Oregon Green BAPTA-1 AM (OGB-1) (Invitrogen, 8 μg/ml (made from a 2.5 μg/μl DMSO stock), containing 0.013% pluronic F-127, (Invitrogen, diluted from a 20% DMSO stock) in pH 7.4, 1 h, room temperature) as we previously described [9]: 10 ml of pH and treatment solutions were introduced into the culture dish at a rate of 100 μl/s through a syringe pump (Harvard Apparatus PHD2000, Holliston, MA). Solution was simultaneously removed from the opposite side of the dish by vacuum aspiration.

Fluorescence was measured on a 150-s time course before, during, and after application of pH solutions. An Olympus IX81 motorized inverted microscope and integrated Intelligent Imaging Innovation’s Slidebook software, v.4.1 was used to capture images. Analysis was done using Image J (NIH) to measure the change of intensity in a constant, defined area of the cell during each pH application. Each session started with pH 7.4 infusion for 20 s, followed by 2 minutes of acidic pH. Between pH solutions, pH 7.4 was again infused. All pH solutions were applied in increasing acidic pH to each culture dish with pH 7.4 infused between each solution. Percent change in (Ca2+)_i_ was quantified using the formula:

$$\%\Delta_{F}=\left(F‒F_{0}\right)/F_{0}*100,$$

where F is the fluorescence intensity at any given time point and F_0_ is fluorescence intensity under control conditions (pH = 7.4, calculated by averaging the intensities recording during the first 20s of each time course). The area under the curve during the 2-minute time period was calculated using the percent-change scores; zero represented no change in area and a positive number represented an increase in calcium intensity.

For Ca^2+^-free pH solutions calcium chloride was eliminated from the external buffers and NaCl adjusted osmolarity to 123 mM. For (Ca2+)_i_ blockade, 30 μM cyclopiazonic acid (CPA, Sigma-Aldrich) was added to the cultures after OGB-1 loading, 5 minutes prior to Ca^2+^ imaging (12 μM CPA was included in treatment during Ca^2+^imaging). In the IL-1β study, 1 ng/ml IL-1β (R and D Systems, Minneapolis, MN, USA) was included in 0.5% FBS serum-starved media 24 h prior to Ca^2+^ imaging.

Two cell culture lines were used for testing FLS from the WT, *ASIC3-/-* and *ASIC1-/-*, Ca^2+^source study, and the IL-1β study. Two cell cultures lines from control (for example, WT) and experimental groups (for example, *ASIC1-/-*) were always run on the same day to control for differences due to loading or imaging. The profiles of percent change (% Δ) as a function of time for all viable cells in a group were averaged and compared, taken as a percent change from the WT average at pH 5 or compared to maximum intensity for each cell.

Amiloride (100, 250, 500 μM; Sigma) was added to cultured human FLS, after OGB-1 loading, 10 minutes prior to and during calcium imaging. Percent change in (Ca2+)_i_ was quantified using the formula:

$$\%\Delta_{F}=\;\left(F‒F_{0}\right)/F_{0}*100,$$

where F is the fluorescence intensity at any given time point and F_0_ is fluorescence intensity under control conditions. Three cell culture lines were used for RA FLS study under each condition.

### Quantitative real-time polymerase chain reaction (qPCR)

FLS were plated at 100,000 cells/well in a 6-well tissue culture plate and grown for 24 h in 10% FBS/DMEM at 37°C with 5% CO_2_ and subsequently incubated in 0.5% FBS/DMEM for 24 h with or without 2 ng/ml IL-1β (Calbiochem, San Diego, CA, USA). FLS were then incubated in 0.5% FBS/DMEM pH 7.4 or pH 6.0 for 24 h. Cells were then lysed in RNA STAT-60 (Tel-Test, Friendswood, TX, USA). FLS RNA isolation and qPCR was performed as previously described [31]. Using cDNA, mRNA expression of IL-6, metalloproteinases (MMPs) and ASICs was measured by TaqMan Gene Expression Assay (Applied Biosystems). The threshold cycle (Ct) values were used to calculate the number of cell equivalents using a standard complementary DNA curve as previously described [31]. The data were normalized to the expression of HPRT (hypoxanthine guanine phosphoribosyltransferase) and the results were expressed as relative expression units. We examined mRNA expression for IL-6, MMP-3 and MMP-13 from WT and *ASIC3-/-* FLS with and without treatment with IL-1β (n = 3 preparations/group), and for ASIC3 and ASIC1 in WT FLS with and without IL-1β at pH 7.4 (n = 4 preparations/group). Different cell culture lines were used for each group (for example, WT, *ASIC3-/-*).

### Western blot analysis

FLS were plated in six-well plates at 200,000 cells/well, and grown 24 h and then incubated in 0.1% FBS/DMEM for 24 h before addition of stimulating factors. Western blot analysis was performed as described previously [5]. After treatments (see below), protein was extracted with lysis buffer (50 mM HEPES, 150 mM NaCl, 25 mM MgCl_2_, 1 mM EDTA, 10% Glycerol, 1% tritonX-100, 20mMβ-glycerophosphate, 10 mM NaF, 1 mM Na_3_VO_4_, supplemented with Complete Proteinase inhibitors (Roche Applied Science, Indianapolis, IN, USA)) and protein concentration determined using the Micro BCA Protein Assay Kit (Thermo Scientific, Rockford, IL, USA). Samples containing 25 μg of protein were resolved on Invitrogen NuPAGE 4% to 12% Bis Tris gels and transferred to a polyvinylidene fluoride (PVDF) membrane. The membranes were blocked with 5% non-fat milk, incubated with primary Ab (all from Cell Signaling Technology, Boston, MA, USA) at 4°C overnight, followed by horseradish peroxidase-conjugated secondary Ab for 1 h. Membranes were developed and imaged using the UVP Bioimaging systems and density of the band was quantified using Image J software (NIH). β-actin was used as a loading control and used as an internal control, and all blots were normalized to β-actin. Data were normalized to the response at pH 7.4. All western blot data analyzed included controls and experimental data for each condition. Data are expressed as the ratio compared to the control. Each blot contained three replicates of control and three replicates of experimental conditions. Each control and experimental condition was run in three separate primary cell cultures for a total of nine preparations for each condition (n = 9/condition).

### Live/Dead assays

FLS were plated onto 12 mm poly-L-lysine-coated round coverslips in 24-well tissue culture plates at 15,000 cells/well and incubated for 24 h in DMEM complete medium at 37°C in an atmosphere of 5% CO_2._ FLS were then incubated for 24 h in 0.5% FBS/DMEM serum starved medium IL-1β, 1 ng/ml.

To determine Ca^2+^ dependency for cell death, FLS were treated 1 h at 37°C in 0.5% FBS/DMEM with or without BAPTA-AM (1 μM, pH 7.4), and then treated for 24 h in 0.5% FBS/DMEM of pH 7.4 or pH 6.0. To determine if p-ERK contributes to the cell death, FLS were treated 1 h with PD98059 (10 μM, pH 7.4) and then 24 h in 0.5% FBS/DMEM of pH 6.0 with PD98059 (10 μM).

Live/Dead assays (Invitrogen) were performed as described previously [1] using a Live/Dead Viability/Cytotoxicity kit (L-3224; Invitrogen), 0.5 mM calcein AM, and 0.5 mM ethidium homodimer 1. Stained cells were then mounted on slides with aqueous CMF-1 mounting medium (Electron Microscopy Sciences, Hatfield, PA)) and imaged with an Olympus BX51 fluorescence microscope with a spot camera. Image J software (NIH) was used to merge and quantify live and dead cells. Data are expressed as the percentage of dead cells. Each experimental condition was analyzed in three different primary cell cultures. An average number of 713 ± 28 cells were counted for each primary cell culture in each condition.

### Statistical analysis

Data are expressed as mean ± standard error of the mean (SEM). (Ca2+)_i_ in response to different pH in FLS from WT, *ASIC1-/-* and *ASIC3-/-* FLS were analyzed by repeated-measures analysis of variance (ANOVA) followed by Tukey’s post hoc test for differences between groups. Differences in Ca^2+^imaging, western Blots, qPCR, and Live-Dead assays for individual experiments were analyzed with one-way ANOVA followed by Tukey’s post-hoc test for group differences. *P*-values less than 0.05 were considered significant.

## Results

### Acidic pH increases (Ca2+)_i_ in FLS

**Figure 1:**
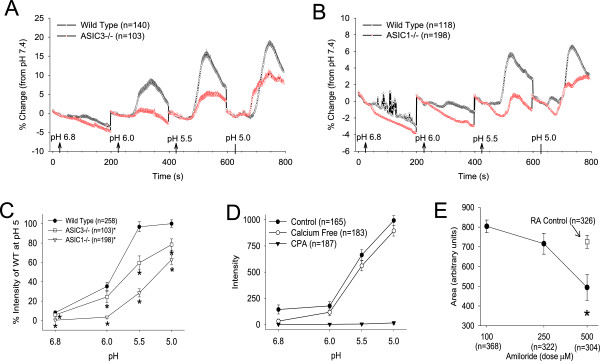
**Ca**^**2+ **^**imaging determines the responses of (Ca2+)**_**i **_**to acidic pH in fibroblast-like synoviocytes (FLS).** (Ca2+)_i_ intensity was calculated for each pH over a 150-s application and normalized as a percent change to the response to pH 7.4. **(A)** (Ca2+)_i_ intensity normalized to pH 7.4 for decreasing pH concentrations in wild-type (WT) and acid-sensing ion channel (*ASIC3-/-*) FLS. Notice a progressive increase in (Ca2+)_i_ intensity with decreasing doses of pH. (Ca2+)_i_ intensity from *ASIC3-/-* run simultaneously showed reduced (Ca2+)_i_ intensity with acidic pH. **(B)** (Ca2+)_i_ intensity normalized to pH 7.4 for decreasing pH concentrations in WT and *ASIC1-/-* FLS. (Ca2+)_i_ intensity from *ASIC1-/-* FLS run simultaneously showed reduced (Ca2+)_i_ intensity with acidic pH. **(C)** Data for *ASIC3-/-* and *ASIC1-/-* were normalized to the (Ca2+)_i_ of WT cells at pH 5.0 to control for variability between cells and between days. A dose-dependent increase in (Ca2+)_i_ occurred in both WT, *ASIC3-/-* and *ASIC1-/-* FLS. However, when compared to WT FLS, *ASIC3-/-* and *ASIC1-/-* FLS showed significantly reduced (Ca2+)_i_ at all pH’s tested when compared to WT FLS (*P <0.05). **(D)** In WT FLS, replacement of the extracellular bath with a Ca^2+^-free medium had no effect on the increases in (Ca2+)_i_ to acidic-pH. Blockade of (Ca2+)_i_ stores with cyclopiazonic acid (CPA) (30 μM) completely abolished the (Ca2+)_i_ increases produced by acidic pH (*P <0.05). **(E)** (Ca2+)_i_ in FLS from people with rheumatoid arthritis show an increase in response to pH 6.0 when compared to responses at pH 7.4 (RA control). This increase in (Ca2+)_i_ response to pH 6.0 is significantly reduced by the non-specific ASIC blocker amiloride (500 μM) (*P* <0.05).

To determine if decreases in pH activate FLS, we performed a pH-response curve from WT FLS (n = 258 cells). Figure 1A and B shows the traces of all cells averaged across time from WT FLS normalized to pH 7.4. When compared to responses at pH 7.4, pH 5.0 produced the maximal increases in (Ca2+)_i_ (944 ± 53 arbitrary units). Increases in (Ca2+)_i_ at increasing pH were progressively smaller, with pH 5.5 showing near peak response (938 ± 64), pH 6.0 showing a moderate response (420 ± 57), and pH 6.8 showing minimal responses (82 ± 23) (Figure 1C).

### ASIC1 and ASIC3 contribute to acidic pH-induced (Ca2+)_i_ increase

To determine the contribution of *ASIC3-/-* and *ASIC1-/-* to the increases in (Ca2+)_i_, we performed a pH-dose response curve in FLS from *ASIC3-/-* (n = 103 cells) and *ASIC1-/-* (n = 198 cells) mice. Figure 1A and B shows the traces of all cells averaged across time from *ASIC3-/-* and *ASIC1-/-* FLS in comparison to WT FLS analyzed under the same conditions. As individual cells show a variation in intensity, the response from knockouts was normalized to peak response observed in WT FLS at pH 5.0. As shown in Figure 1C there was a significant reduction in (Ca2+)_i_ intensity at all pH’s tested in both *ASIC3-/-* and *ASIC1-/-* FLS.

The contribution of intracellular or extracellular Ca^2+^ to the increases in (Ca2+)_i_ produced by decreases in pH was then examined by evaluating decreasing pH in WT FLS in a Ca^2+^-free medium (n = 183 cells) or after depletion of Ca^2+^ stores with the sarco-endoplasmic reticulum Ca^2+^-ATPase (SERCA) inhibitor, CPA (n = 187 cells). Similar increases in (Ca2+)_i_ occurred in WT FLS with and without extracellular Ca^2+^ in the bath (Figure 1D). On the other hand, pretreatment using CPA completely abolished the increases in (Ca2+)_i_ produced by acidic pH (Figure 1B). Thus, the (Ca2+)_i_ increases in response to decreases in pH through ASICs occur by release from (Ca2+)_i_ stores.

To confirm that human FLS also respond to acidic pH in an ASIC-dependent manner, we examined the (Ca2+)_i_ intensities of RA FLS after treatment with pH 6.0 with and without the non-selective ASIC blocker amiloride (100 μM n = 368 cells; 250 μM n = 322 cells; 500 μM n = 304 cells; controls, n = 326 cells). pH 6.0 significantly increased (Ca2+)_i_ intensity in RA FLS (Figure 1E). Pre-treatment of the FLS with amiloride reduced this increase in (Ca2+)_i_ in a dose-dependent manner (Figure 1C). A significant reduction in the amplitude of pH 6.0-induced (Ca2+)_i_ response occurred with 500 μM amiloride (*P* <0.05). Thus, ASICs mediate (Ca2+)_i_ increases induced by acidic pH.

### Acidic pH decreases p-ERK protein expression in FLS

**Figure 2:**
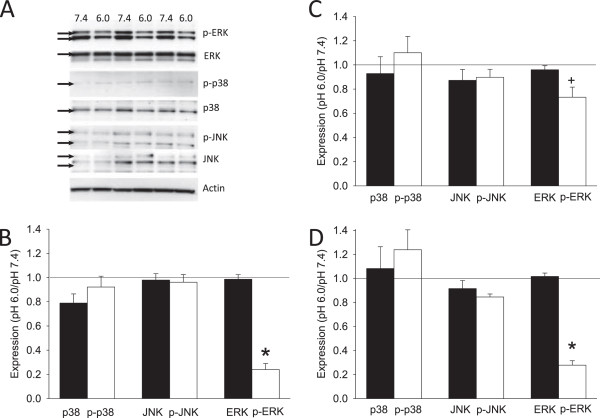
**Western blot analysis determines the responses of mitogen-activated protein kinases (MAPKs) after incubation in pH 6.0 or 7.4 in wild-type (WT), acid-sensing ion channel *(ASIC3)-/- *and *ASIC1-/- *fibroblast-like synoviocytes (FLS). (A)** Representative western blots for extracellular signal-regulated kinase (ERK), p-ERK, p-p38, p-38, c-Jun N-terminal kinase (JNK), p-JNK and actin treated with pH 7.4 or pH 6.0. Blots were always run with pH 7.4 or pH 6.0 simultaneously. Notice the decreases in p-ERK with pH 6.0 when compared to pH 7.4. The bands that were quantified for each protein are highlighted with an arrow and are previously described by us [31]. **(B)** WT FLS: density of the individual bands was taken using Image J software and pH 6.0 was normalized to pH 7.4; pH 6.0 and pH 7.4 were always run simultaneously on the same blots to facilitate comparison between groups. Expression values of 1.0 represent no change, <1.0 represent a decrease in expression, and > 1.0 represent an increase in expression. p-ERK protein expression significantly decreased after exposure to pH 6.0 in WT FLS when compared to the response at pH 7.4 (*P <0.05). **(C)***ASIC3-/-* FLS: p-ERK protein expression was significantly greater 15 minutes after exposure to pH 6.0 in *ASIC3-/-* FLS when compared to WT FLS (^+^*P* <0.05). **(D)***ASIC1-/-* FLS: p-ERK protein expression significantly decreased after exposure to pH 6 in *ASIC1-/-* FLS when compared to the response at pH 7.4 (*P <0.05), and was similar to the decreased expression observed in WT FLS.

MAPKs play a significant role in mediating the inflammatory response by synoviocytes [16-20]. Therefore, we tested if MAPKs were modulated by acidic pH using western blot analysis. WT FLS were incubated in pH 6.0, and compared to pH 7.4, (WT, n = 9 preparations/condition). Unexpectedly, p-ERK protein expression significantly decreased 15 minutes after exposure to pH 6 in WT FLS when compared to the response at pH 7.4 (*P* <0.05) (Figure 2A and B). No changes were observed in total ERK, or in p-JNK, JNK, p-p38, or p-38 (Figure 2A and B).

We then examined the contribution of ASICs to the acid-induced decrease in p-ERK using *ASIC3-/-* (n = 9/condition) and *ASIC1-/-* FLS (n = 9/condition). The decrease in p-ERK expression induced by pH 6.0 did not occur in *ASIC3-/-* FLS and was significantly greater than WT FLS treated with pH 6.0 (*P* <0.05) (Figure 2C). On the other hand, p-ERK expression significantly decreased in *ASIC1-/-* FLS (Figure 2D). This decrease was similar to that observed in WT FLS treated with pH 6.0 (*P* >0.05). Thus, the decreases in p-ERK induced by acidic pH require ASIC3, but not ASIC1.

### Role of (Ca2+)_i_ in regulation of p-ERK by acidic pH

**Figure 3:**
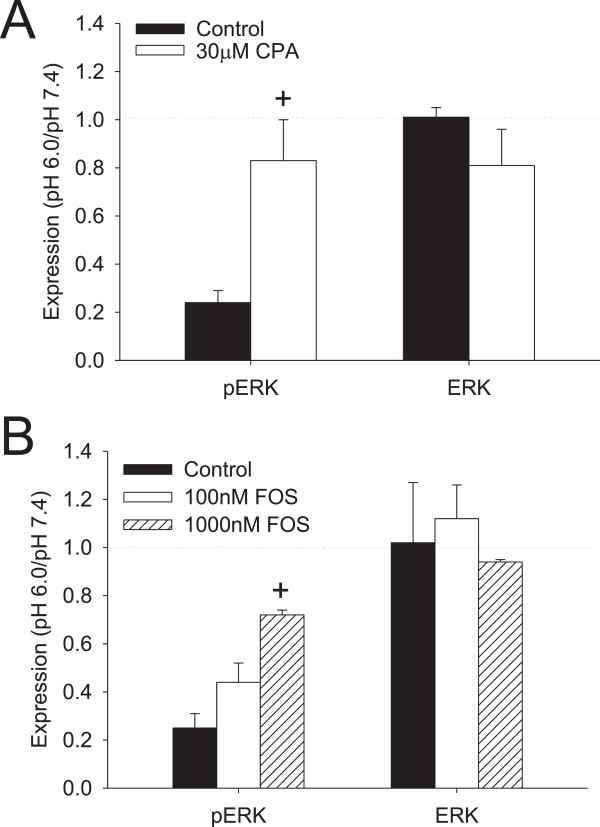
**Western blot analysis determines the responses of extracellular signal-regulated kinase (ERK), p-ERK and in wild-type (WT) fibroblast-like synoviocytes (FLS) to blockade of (Ca2+)**_**i **_**with cyclopiazonic acid *(*CPA) and of protein phosphatase 2A (PP2A) with fostriecin (FOS).** Responses after incubation in pH 6.0 were normalized to pH 7.4. **(A)** Pretreatment of WT FLS with CPA (30 μM) prior to application of pH 6.0 prevented the decrease in p-ERK when compared to WT FLS not treated with CPA (control) (*P* <0.05). **(B)** Blockade of PP2A with FOS (1000 nM) prevented the decrease in p-ERK in WT FLS treated with pH 6.0 when compared to WT FLS without FOS treated with pH 6.0 (control) (*P* <0.05).

As acidic pH increases (Ca2+)_i_ and decreases p-ERK, we tested if blockade of (Ca2+)_i_ stores prevented the decrease in p-ERK expression in WT FLS (n = 9 preparations/condition). Pretreatment of FLS with CPA (30 μM) prior to and during application of pH 6.0 prevented the decrease in p-ERK in WT FLS (Figure 3A) (*P* <0.05). There were no changes in ERK expression with CPA treatment. As protein phosphatase 2A (PP2A) can modulate ERK activity [27] and is activated by increases in (Ca2+)_i_, we then determined if PP2A blockade prevents the acidic pH-induced decreases in p-ERK. Inhibition of PP2A with fostriecin (FOS) (100 and 1,000 nM) dose-dependently prevented the decrease in p-ERK in WT FLS treated with pH 6.0 (Figure 3B). A significant difference from those treated with 1,000 nM FOS occurred when compared to control FLS not treated with FOS (*P* <0.05).

### Effects of the IL-1β on pH-induced changes in FLS

**Figure 4:**
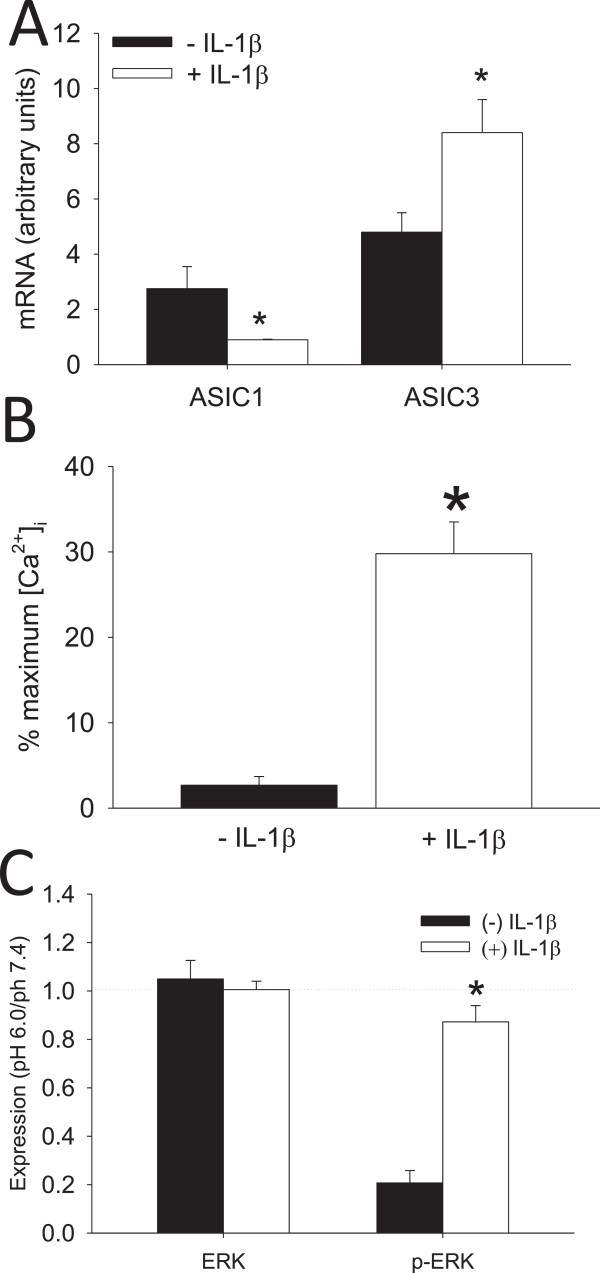
**Effects of the inflammatory mediator IL-1β on acid-induced changes in wild-type (WT) fibroblast-like synoviocytes (FLS). (A)** Real-time PCR of WT FLS determined the expression of acid-sensing ion channel (ASIC)1 and ASIC3 after incubation with or without IL-1β at pH 7.4. There was a significant increase in expression of ASIC3 mRNA and a decrease in expression of ASIC1 mRNA in the group treated with IL-1β when compared to WT FLS not treated with IL-1β (*P* <0.05). **(B)** Ca^2+^ imaging determined (Ca2+)_i_ in WT FLS treated with IL-1β followed by exposure to pH 6.0 and normalized to responses at pH 7.4. WT FLS incubated with IL-1β showed greater increases in (Ca2+)_i_ intensity when compared to WT FLS not treated with IL-1β (*P* <0.05). **(C)** Western blot analysis determined the acid-induced responses of extracellular signal-regulated kinase (ERK) and p-ERK in WT FLS treated with IL-1β. After exposure to pH 6.0, p-ERK expression was significantly higher in WT FLS with IL-1β when compared to that in WT FLS without IL-1β (*P* <0.05).

Previous studies show an upregulation of ASICs after inflammation in sensory neurons [4,32,33]; however, whether this upregulation occurs in other cells like FLS is unknown. To mimic the inflammatory conditions observed *in vivo*, we incubated FLS with the inflammatory cytokine IL-1β and examined expression of ASIC3 and ASIC1 mRNA using qPCR (n = 4 preparations/group). There was a significant increase in expression of ASIC3 mRNA in FLS treated with IL-1β when compared to control FLS (*P* <0.05). In contrast, there was a simultaneous decrease in expression of ASIC1 in WT FLS treated with IL-1β (Figure 4A) (*P* <0.05). Thus, IL-1β significantly increased expression of ASIC3 in FLS.

To determine whether there was enhanced (Ca2+)_i_ in WT FLS sensitized by IL-1β we incubated FLS in IL-1β followed by exposure to pH 6.0 (n = 112 cells); all responses were normalized to responses to pH 7.4 in the same cells. FLS exposed to the inflammatory mediator IL-1β demonstrated a significantly greater increase in (Ca2+)_i_ intensity to pH 6.0 when compared to cells not treated with IL-1β (Figure 4B)(*P* <0.05). Thus, IL-1β enhanced the acid-induced increase in (Ca2+)_i_.

To determine whether acid-induced decreases in p-ERK are modified in cells sensitized by IL-1β, we examined the effects of pH 6.0 on p-ERK protein in WT FLS treated with IL-1β (n = 9 preparations/condition). p-ERK expression in response to pH 6.0 was significantly higher in WT FLS treated with IL-1β, when compared to that observed in WT FLS without IL-1β (Figure 4C) (*P* <0.05). The role of ASICs in the p-ERK changes was then explored using *ASIC1-/-* and ASC3-/- FLS. Interestingly, a significant decrease in p-ERK in response to pH 6.0 occurred after pre-treatment with IL-1β in both *ASIC1-/-* (0.16 ± 0.02, n = 9) FLS and *ASIC3-/-* (0.26 ± 0.05, n = 9) FLS (*P* <0.05). No changes in ERK (Figure 4C), JNK, p-JNK, p38, or p-p38 (data not shown) were observed in WT, *ASIC3-/-* or *ASIC1-/-* FLS treated with IL-1β and pH 6.0. Thus, the enhanced p-ERK expression observed in cells sensitized by IL-1β was mediated by ASIC1 and ASIC3.

### Regulation of FLS gene expression by acidic pH in FLS

As our prior study in *ASIC3-/-* mice showed increases in IL-6, MMP-3 and MMP-13 gene expression in the ankle joint of arthritic mice when compared to WT mice [1], we tested whether acidic pH and ASIC3 modulate gene expression of these inflammatory mediators in FLS. In WT FLS treated with IL-1β, there was a significant increase in mRNA expression for IL-6, MMP-3 and MMP-13 compared to WT FLS without IL-1β treatment. FLS treated with pH 6.0 showed no difference from those treated with pH 7.4, with or without IL-1β. *ASIC3-/-* FLS showed similar increases in mRNA expression of IL-6, MMP-3 and MMP-13 after exposure to IL-1β and were not significantly different from WT FLS (Table 1). Thus, acidic pH and ASIC3 do not modulate the enhanced expression of IL-6, MMP-3 and MMP-13 induced by IL-1β.

**Table 1:** **Real-time PCR determines the gene expression of cytokines in WT FLS and***ASIC3-/-***FLS after incubation in pH 6.0 or 7.4 with or without IL-1β**

**Cytokine**	**Il-1b treatment**	**pH 7.4**		**pH 6.0**	
		**WT**	***ASIC3-/-***	**WT**	***ASIC3-/-***
**IL-6**	(-)	0.0018 ± 0.0009	0.0007 ± 0.0002	0.0037 ± 0.0028	0.001 ± 0.0004
	(+)	0.0886 ± 0.0218	0.1136 ± 0.013	0.1488 ± 0.0503	0.1829 ± 0.0411
**MMP-3**	(-)	0.0307 ± 0.0042	0.0180 ± 0.0060	0.0373 ± 0.0072	0.0207 ± 0.0097
	(+)	1.7975 ± 0.7027	1.7750 ± 0.712	1.81 ± 0.7002	1.8055 ± 0.6042
**MMP-13**	(-)	0.0642 ± 0.0062	0.0677 ± 0.0308	0.0761 ± 0.006	0.0879 ± 0.0526
	(+)	1.5977 ± 0.4506	1.5066 ± 0.5933	1.8362 ± 0.5362	1.5015 ± 0.5819

mRNA expression of IL-6, metalloproteinase (MMP)-3 and MMP-13 all significantly increased in wild-type (WT) fibroblast-like synoviocytes (FLS) and acid-sensing ion channel (ASIC3)-/- FLS treated with IL-1β when compared to FLS without IL-1β. There were no differences between WT FLS treated with pH 6.0 and those treated pH 7.4, and there was no difference between WT FLS and *ASIC3-/-* FLS. Results are presented as mean ± standard error of the mean.

### Regulation of apoptosis by acidic pH

**Figure 5:**
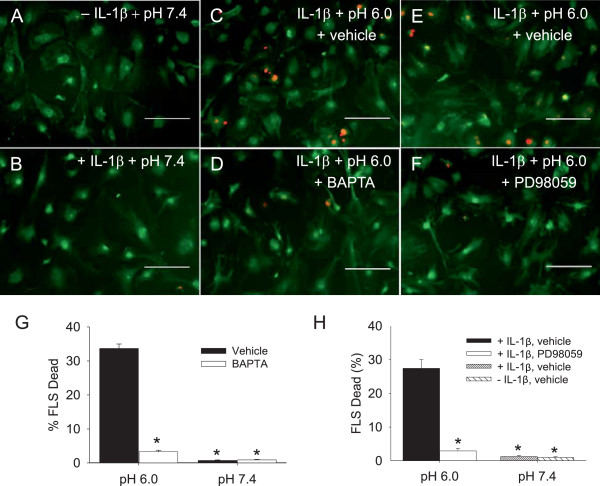
**Live/Dead assays determined calcium dependency and extracellular signal-regulated kinase (ERK) contribution to cell death induced by pH 6.0 with IL-1β.** The number of dead fibroblast-like synoviocytes (FLS) and the number of live FLS were counted to obtain the percent dead. **(A-F)** Representative images from FLS stained using a Live/Dead Viability/Cytotoxicity kit. Live cells were stained with calcein AM (green), dead cells were stained with ethidium homodimer (red). **(A and B)** Show images of FLS treated by pH 7.4 without or with IL-1β, respectively. **(C and D)** Show images of FLS treated by pH 6.0 and IL-1β without or with intracellular calcium chelating agent (BAPTA, 1 μM), respectively; **(E and F)** show images of FLS treated by pH 6.0 and IL-1β without or with inhibitor of ERK (PD98059,10 μM), respectively. **(G)** Quantitative analysis shows approximately 30% dead FLS after treatment with pH 6.0 and IL-1β, which was significantly greater than FLS treated with pH 6.0 and IL-1β with BAPTA, and pH 7.4 and IL-1β with and without BAPTA (*P <0.01). **(H)** Quantitative analysis shows approximately 30% dead FLS after treatment with pH 6.0 and IL-1β, which was significantly greater than FLS treated with pH 6.0 and IL-1β with PD98059 (10 μM), and FLS with or without IL-1β and pH 7.4 (*P <0.001). Data are mean ± standard error of the mean.

We previously showed that pH 6.0 in combination with IL-1β enhanced synoviocyte cell death in FLS from WT mice when compared to IL-1β alone, pH 6.0 alone, or pH 7.4, and this enhanced cell death depended on ASIC3 [14]. As increases in (Ca2+)_i_ can induce cell death, and ASIC3 mediates the acid-induced increases in (Ca2+)_i__,_ we tested whether the acid-induced cell death depends on (Ca2+)_i_ in WT FLS. Representative images in Figure 5 show that cell death increased in WT FLS exposed to pH 6.0 and IL-1β when compared to those exposed to pH 7.4 with and or without IL-1β. After exposure of WT FLS to IL-1β and pH 6.0, there were significantly fewer dead cells in the group incubated with BAPTA-AM to chelate (Ca2+)_i_ when compared to vehicle (Figure 5C,D,G). No changes in cell death occurred in FLS treated with pH 7.4 with or without IL-1β (Figure 5A,B,G,H).

As we showed that acid-induced decreases in p-ERK are modulated by increases in (Ca2+)_i_, and prior studies show that inhibiting ERK attenuates cell death [25], we tested whether blockade of ERK would prevent the acid-induced cell death. FLS treated with PD98059 displayed significantly less death in response to IL-1β and pH 6.0 when compared to WT FLS controls (pH 6.0 + IL-1β) (Figure 5E,F,H). No changes in cell death occurred in FLS treated with pH 7.4 and IL-1β with or without PD9805 (Figure 5C). Thus, (Ca2+)_i_ and ERK modulate the enhanced cell death that occurs in response to acidic-pH under inflammatory conditions.

## Discussion

The current study demonstrated a unique role for ASIC3 in FLS in modulating (Ca2+)_i_, phosphorylation of the MAP kinase ERK, and cell death induced by acidic pH. Specifically, we showed that activation of ASIC3 by acidic-pH increases (Ca2+)_i_ in FLS, which subsequently reduces phosphorylation of ERK and this reduced p-ERK is prevented by blockade of PP2A. After sensitization of FLS with IL-1β, which mimics inflammatory synovitis: 1) acidic pH further enhances (Ca2+)_i_; 2) acid-induced decreases in p-ERK are abolished, and 3) acidic pH induces apoptosis. The cell death that occurs in IL-1β-treated FLS exposed to acidic pH is blocked by chelation of (Ca2+)_i_, inhibition of ERK, and does not occur in *ASIC3-/-* FLS [1]. Further, there is enhanced inflammation and joint degradation in *ASIC3-/-* mice with passive collagen-induced arthritis (CAIA) [1]. Taken together these data support a protective role of ASIC3 under inflammatory conditions - activation of ASIC3 would cause cell death and limit synovitis.

### Role of ASICs in (Ca2+)_i_ increases in FLS

Our data agree with prior studies showing that acidic pH increases (Ca2+)_i_[1,9,34,35]. In agreement with our prior study [9], ASIC3 mediates these increases. We further show that genetic deletion of ASIC1 also reduced the (Ca2+)_i_ produced by acidic pH. Interestingly, previous studies show increases within the same pH range as the present study, pH 5.5 or with pH 6.8; however direct comparison of (Ca2+)_i_ responses between differing acidic pH was not previously examined, and these studies were done from FLS taken from animals with CAIA [35,36]. In the current study we did not eliminate all (Ca2+)_i_ increases in FLS from *ASIC3-/-* or from *ASIC1-/-*. As ASICs form heteromers [5,37], TRPV1 can respond to acidic pH [38,39], and a G-proton-sensing acid channel was previously identified in FLS [40], it is likely that removal of any one channel would not eliminate the acid-response. The overall abundance of TRPV1 (transient receptor potential cation channel vanilloid 1) is relatively low in comparison to ASIC3 in FLS from WT mice [9] suggesting a larger contribution of proton response occurs for ASIC. Interestingly, capsaicin-evoked increases in (Ca2+)_i_ were actually reduced at pH 6.8 suggesting another channel or process inhibits the TRPV1 channel. Since each ion channel might have different intracellular targets, their response to acid could be unique. Thus, ion channels located on FLS can control levels of (Ca2+)_i_ and are activated and modulated by decreases in pH.

### Role of ASICs in modulation of p-ERK

Decreased ERK phosphorylation in response to acidic pH in WT FLS was surprising, as MAPKs enhance inflammatory cytokine expression in FLS [16-20]. Synoviocytes are key players in the production of inflammatory mediators (for example, IL-6, TNF, MMP), which subsequently enhance the inflammatory process and joint damage [13-17]. Prior studies showed that increased (Ca2+)_i_ enhanced expression of the catalytic subunit of PP2A which decreased ERK phosphorylation [27]. We confirmed the role of calcium and PP2A by showing that the decrease in p-ERK is reduced by blockade of PP2A and (Ca2+)_i_. It is possible that the changes in p-ERK that occur by blockade of PP2A are the result of a direct action of PP2A on p-ERK changes or a parallel pathway that results in increased ERK.

### Effects of acidic pH under inflammatory conditions

The current study shows differences in response to acidic pH in FLS treated with IL-1β, including enhanced (Ca2+)_i_, no changes in p-ERK, and cell death. Exposure to inflammatory mediators such as IL-1β can be used to mimic the inflammatory condition. We recognize there are a number of other key mediators present in arthritic joints, for example, TNF and IL-6, and future experiments should examine their effects on ASICs.

Our prior study in *ASIC3-/-* mice with CAIA showed enhanced swelling as well as enhanced IL-6, MMP-3 and MMP-13 expression in the joint tissue [1] suggesting that ASIC3 might reduce gene expression of these inflammatory mediators. Although IL-1β increased expression of IL-6, MMP-3 and MMP-13 in WT FLS, these changes were similar after application of acidic pH and in *ASIC3-/-* FLS. Thus, we conclude that the enhanced expression of inflammatory mediators in *ASIC3-/-* mice results from excess synoviocyte proliferation that would normally be prevented in WT mice by the activation of ASIC3 on FLS in the acidic environment of an inflamed joint.

Synovial intimal lining hyperplasia is a hallmark of RA and synoviocytes release inflammatory cytokines and MMPs contributing to the inflammation and joint destruction [20,41]. Increasing synoviocyte cell death during synovitis might be a mechanism to control inflammatory joint disease progression. For example, a prior study that expressed human calcineurin binding protein 1 in synoviocytes of mice with CAIA showed enhanced cell death and reduced cytokine and MMP expression [42]. In contrast the endogenous substance vascular endothelial growth factor (VEGF) protects against synoviocyte cell death, is increased in synovial fluid from individuals with RA [43], and blockade of VEGF reduces disease severity in mice with CAIA and enhances cell death in cultured synoviocytes [44]. Thus, we propose that ASIC3 plays a role in limiting synovitis by inducing cell death under conditions of inflammation where the pH of the joint is reduced; this could be a normal healthy response to joint inflammation.

Our prior work shows that *ASIC3-/-* mice have enhanced inflammation and joint degradation, and sensitizing FLS with the inflammatory mediator IL-1β results in cell death to pH 6 that requires ASIC3 [1]. The current study showed that this IL-1β-acid-induced cell death of FLS requires (Ca2+)_i_ increases and activation of ERK. This agrees with prior studies showing that ERK activation can cause cell death in a variety of cell types including neurons, cancer, chondrocytes and macrophages [23-26] - we now show that ERK plays a role in cell death of synoviocytes. In contrast to our study, RA FLS stimulated with VEGF, which protects against cell death, increases p-ERK and this increase in p-ERK is blocked by inhibition of VEGF [45]. ERK activation in synoviocytes could be dependent on the state of the cell, the location of ERK within a cell compartment, the cell surface and intracellular pathways that are connected to ERK, or the ERK subtype activated.

## Conclusions

Inflammation, joint damage, and pain are critical components of RA. The current study suggests that activation of ASIC3 on FLS can modulate inflammation by enhancing synoviocyte cell death and limit synovitis. Thus ASIC3 could be a therapeutic target to control disease progression. In contrast, our previously published work consistently shows that ASIC3 plays a role in the transmission of nociceptive (painful) information in animal models of musculoskeletal pain where there are reduced pain behaviors in *ASIC3-/-* mice or downregulation of ASIC3 in peripheral neurons [1-3,6-8]. Thus, ASIC3 located on nociceptors innervating inflamed tissue appears to produce pain-behaviors while simultaneously through its location on synoviocytes reduces inflammation by causing synoviocyte cell death. Future studies should determine if alterations in ASIC3, activation and blockade, have differential effects in WT animals with inflammatory arthritis.

**Figure 6:**
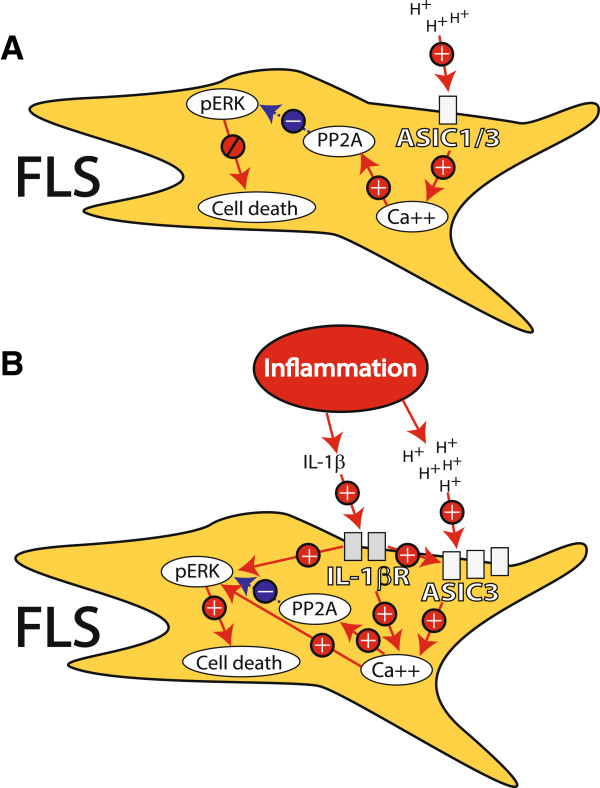
**A schematic of the role of acid-sensing ion channel (ASIC)1 and ASIC3 involved in synoviocyte function under normal and inflammatory conditions. (A)** This diagram shows fibroblast-like synoviocytes (FLS) under normal non inflamed conditions. Extracellular protons (H^+^, acidic pH) activate the ASIC3 and ASIC1 that increase calcium release from intracellular stores. The increases in intracellular calcium through ASIC3 activate protein phosphatase 2A (PP2A) which subsequently dephosphorylates extracellular signal-regulated kinase (ERK). This reduction in phosphorylation of ERK counteracts the increases in intracellular calcium so that there is no cell death of FLS. **(B)** Under inflammatory conditions, FLS are exposed to inflammatory mediators, such as IL-1β as well as an acidic environment. The combination of these two factors results an enhanced response and cell death. IL-1β increases expression of ASIC3 and decreases expression of ASIC1 results in ion channels with a greater proportion of ASIC3 on FLS to sense the increased protons from the acidic environment. Activation of ASIC3 by protons would then result in enhanced calcium release intracellularly. IL-1β by itself can modulate calcium and can enhance phosphorylation of ERK. This enhanced intracellular calcium by activation of ASIC3 and IL-1β, along with enhanced phosphorylation of p-ERK by activation of IL-1β receptor activation shifts the balance of calcium and p-ERK to favor cell death. Thus, under inflammatory conditions, decreases in pH activate ASIC3 to produce cell death.

In normal resting FLS, decreases in pH activate ASIC3 to increase (Ca2+)_i_ activating PP2A, which dephosphorylates ERK and there is no cell death (Figure 6). Decreases in pH also activate ASIC1 to increase (Ca2+)_i_. In synovitis, inflammatory mediators are released that can activate intracellular pathways to increase expression of ASIC3, decrease expression of ASIC1, increase (Ca2+)_i_, and increase phosphorylation of ERK. The consequence of this is an amplified response to acidic pH to result in cell death. Thus, decreases in pH under inflammatory conditions play a protective role by activating ASIC3 to limit synovitis and could be a potential new therapeutic target.

## Abbreviations

ANOVA: analysis of variance; ASIC: acid sensing ion channel; CAIA: collagen-induced arthritis; CPA: cyclopiazonic acid; DMEM: Dulbecco’s modified Eagle’s medium; ERK: extracellular signal-regulated kinase; FBS: fetal bovine serum; FLS: fibroblast-like synoviocyte; FOS: fostriecin; IL: interleukin; JNK: c-Jun N-terminal kinase; MAPK: mitogen-activated protein kinase; MMP: metalloproteinase; OGB-1: Oregon green BAPTA-1-AM; PP2A: protein phosphatase 2A; qPCR: quantitative real time polymerase chain reaction; RA: rheumatoid arthritis; SERCA: sarco-endoplasmic reticulum Ca^2+^-ATPase; TNF: tumor necrosis factor; VEGF: vascular endothelial growth factor; WT: wild-type.

## Competing interests

The authors have no competing financial or non-financial interests directly related to this manuscript.

## Authors’ contributions

WG carried out cell death experiments, wrote an initial draft of the manuscript, and read and revised the manuscript. SK carried out the calcium imaging, was involved in design of all studies, trained WG in cell-death experiments, wrote some methods for the manuscript, and read and revised the manuscript. YU was involved in experimental design for the calcium imaging studies, and read and edited the manuscript. RW was involved in experimental design of the experiments, and developed and trained technical support on western blot analysis. DB carried out the qPCR experiments, was involved in experimental design, and edited the manuscript. GF conceived of the ideas, was involved in experimental design and edited the manuscript. KS conceived of the ideas, was involved in experimental design of all experiments, performed statistical analysis, coordinated the experiments, and wrote and revised the final draft of the manuscript. All authors read and approved the final version of the manuscript.
